# Supplementary material for: Identifying Features of a System of Practice to Inform a Contemporary Competency Framework for Paramedics in Canada
Source: Healthcare (Basel). 2024 May 5;12(9):946. doi: 10.3390/healthcare12090946 (PMC11083595; doi:10.3390/healthcare12090946)
Supplement: Supplementary file 1 [file healthcare-12-00946-s001.zip › healthcare-2955811-supplementary.pdf]

Supplementary File S1 - Diagramming

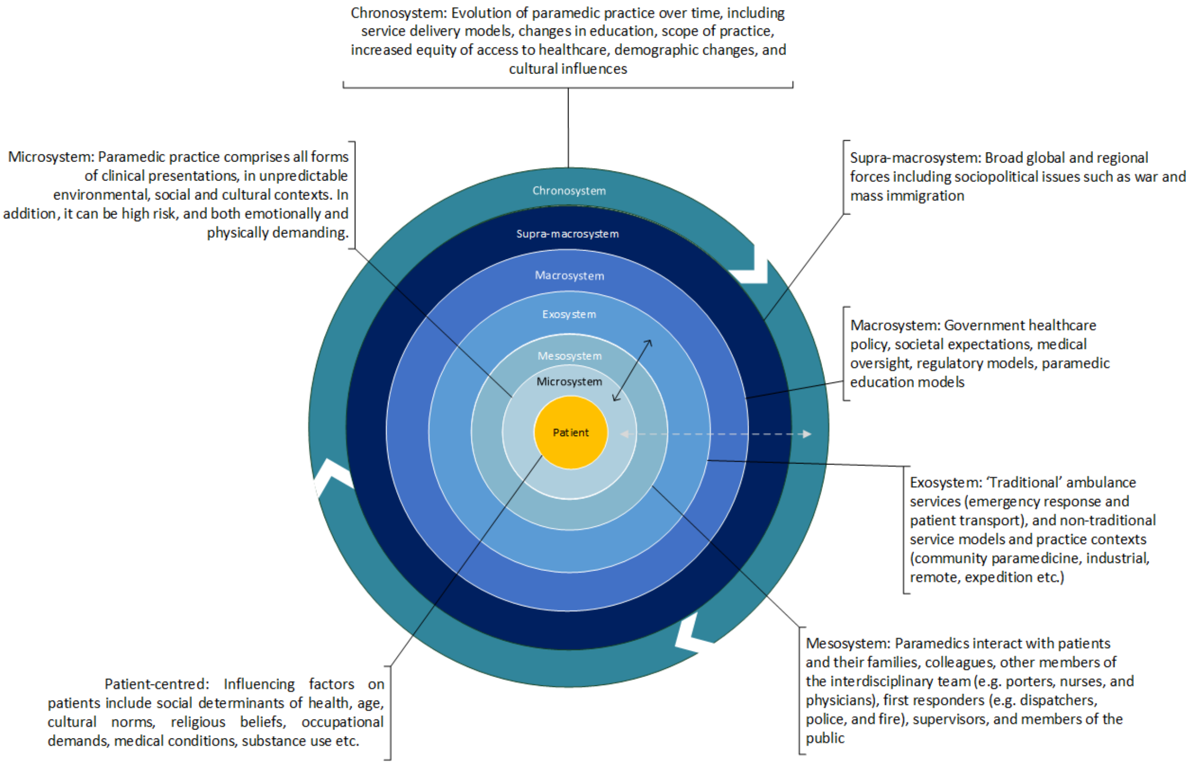

Figure S1. Onion diagram of the system levels of paramedic practice in Canada

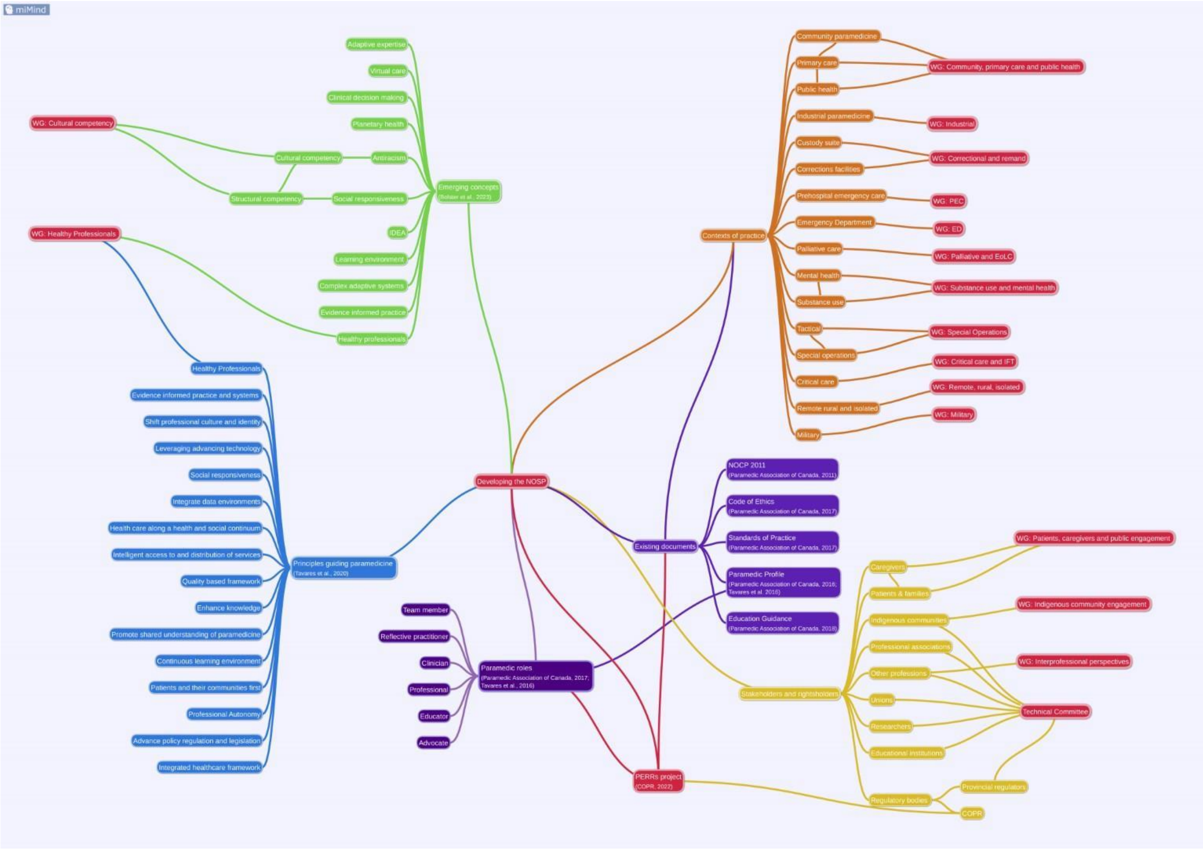

Figure S2 . Concept mapping of the system of paramedic practice in Canada and influences on the development process.

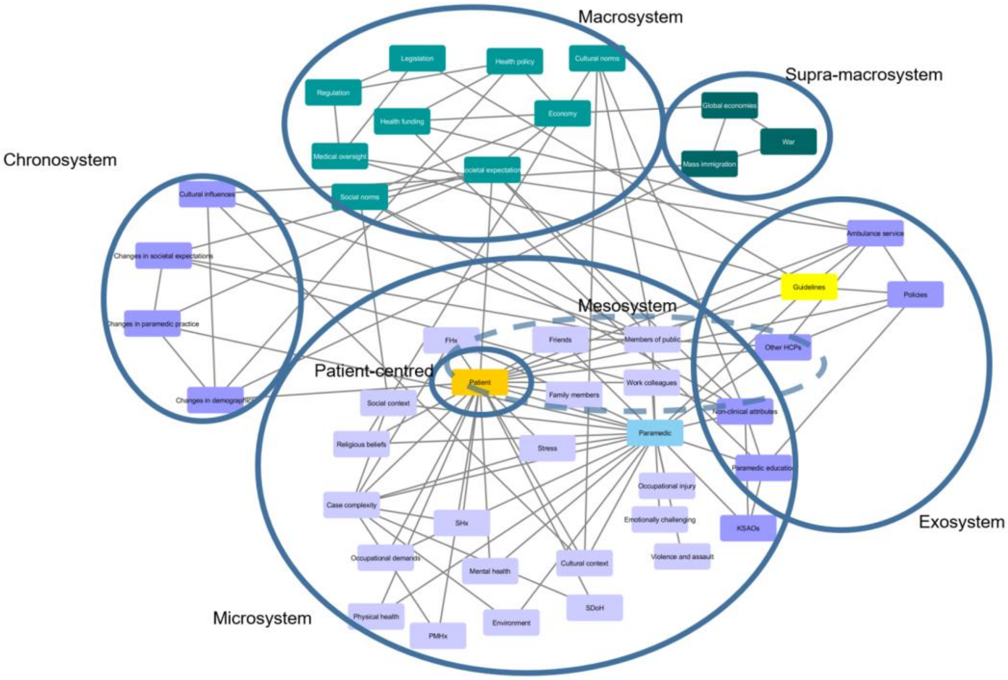

Figure S3. Influence mapping of the system of paramedic practice in Canada.

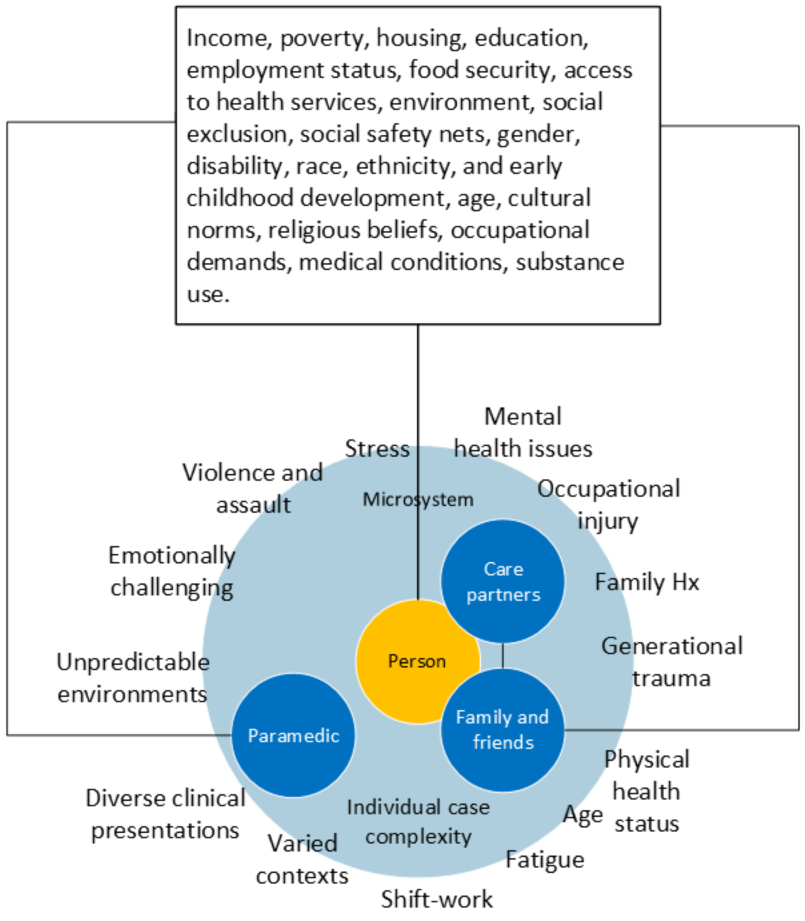

Figure S4. Microsystem details.

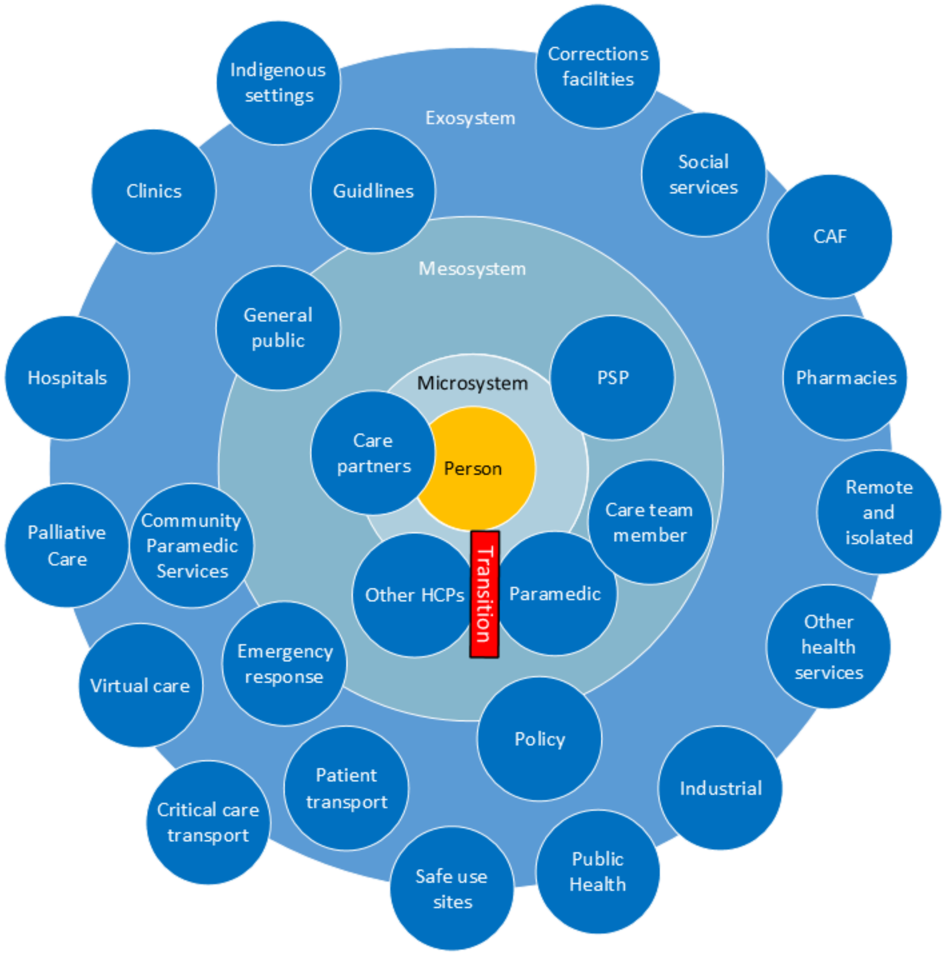

Figure S5. Mesosystem and exosystem details.

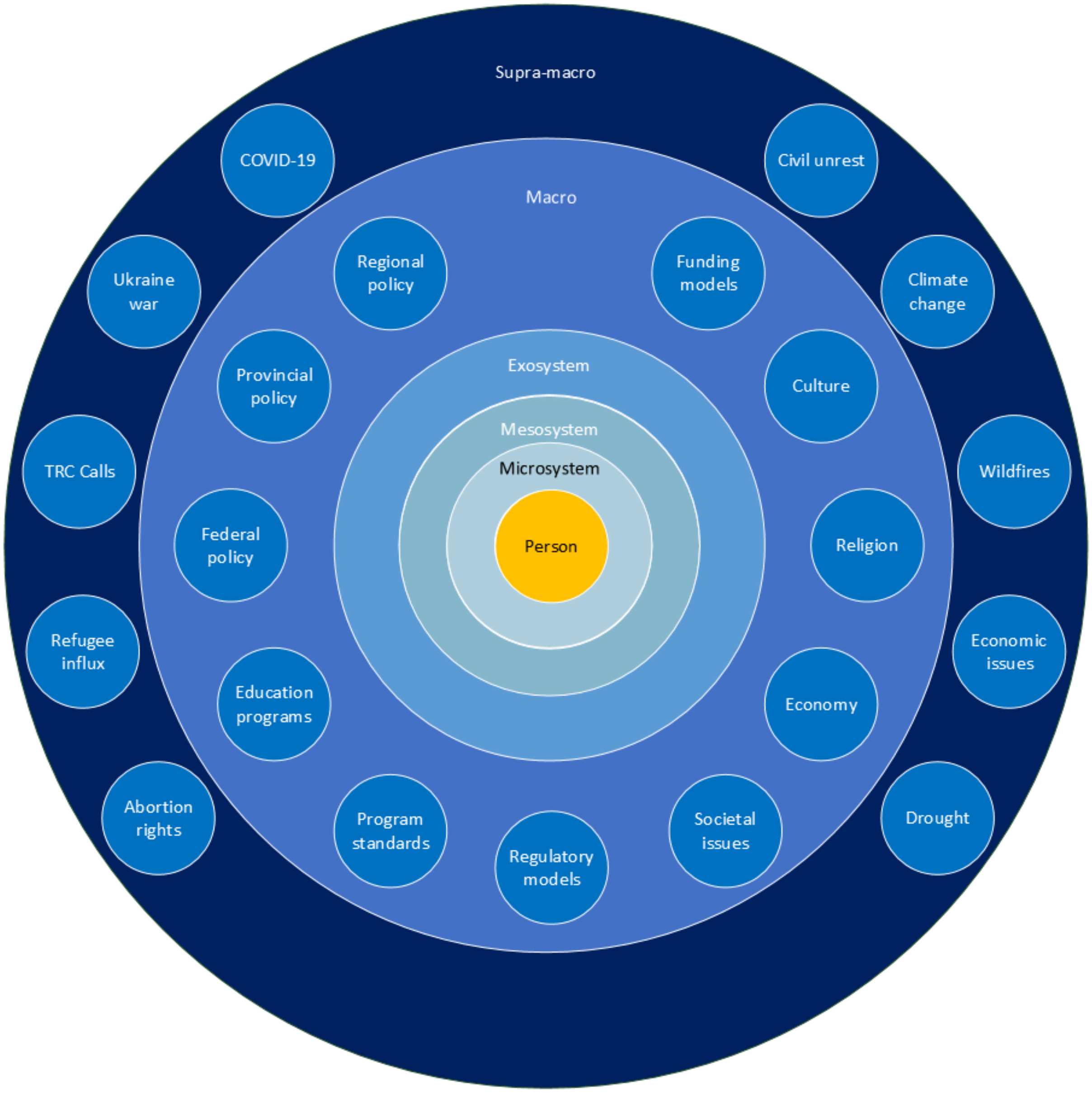

Figure S6. Macrosystem and supramacrosystem details.
